# Contact-Accessible Silver Nanoparticle-Decorated Electrospun Carbon Fibers for Microplastics Detection by SERS

**DOI:** 10.3390/ma19061074

**Published:** 2026-03-11

**Authors:** FNU Joshua, Yuen Yee Li Sip, Aritra Biswas, Violette Gray, Debashis Chanda, Lei Zhai

**Affiliations:** 1 NanoScience Technology Center, University of Central Florida, Orlando, FL 32826, USA; yuenyee.lisip@ucf.edu (Y.Y.L.S.); aritra.biswas@ucf.edu (A.B.); debashis.chanda@ucf.edu (D.C.); 2 Department of Chemistry, University of Central Florida, Orlando, FL 32826, USA; 3 Department of Materials Science and Engineering, University of Central Florida, Orlando, FL 32816, USA; 4 College of Optics and Photonics, University of Central Florida, Orlando, FL 32816, USA; 5 Department of Physics, University of Central Florida, Orlando, FL 32826, USA; 6 Department of Mechanical Engineering, Daytona State College, Daytona Beach, FL 32114, USA

**Keywords:** microwave, mixed-solvent synthesis, silver nanoparticles, electrospun carbon fibers, microplastics, SERS

## Abstract

Reliable detection of microplastics by surface-enhanced Raman scattering (SERS) is often hindered by poor particle–substrate contact and limited access to plasmonic hotspots on conventional planar substrates optimized for molecular adsorption. Here, we report a rapid microwave-assisted carbothermal shock strategy to fabricate silver nanoparticle-decorated electrospun carbon fibers (AgNPs@ECF) as a three-dimensional plasmonic platform tailored for solid microplastic sensing. Localized microwave-induced heating in a mixed ethanol–hexane system enables Ag nanoparticle nucleation and anchoring on conductive carbon fibers within 45 s, yielding a mechanically compliant, junction-rich architecture without chemical reductants or vacuum processing. The AgNPs@ECF composite was evaluated using morphologically weathered polystyrene (PS) and polyethylene terephthalate (PET) microplastics, along with size-controlled PS bead standards ranging from ~50 nm to 45 μm. Across these models, SERS response is governed primarily by particle–substrate contact geometry and near-field accessibility rather than polymer type. The strongest enhancement occurs in the sub-micrometer regime, where particles can engage multiple AgNP-decorated fiber junctions, while ultrasmall and large, smooth particles show reduced enhancement due to limited contact or rapid field decay. Spatially resolved Raman mapping and finite-difference time-domain simulations support a contact-dominated enhancement mechanism, revealing localized field confinement at particle–fiber interfaces. These results establish the design principles for three-dimensional SERS substrates targeting heterogeneous solid particulates, demonstrating that contact-accessible plasmonic architectures are critical for reliable microplastic detection under realistic solid-particle measurement conditions.

## 1. Introduction

Microplastics, broadly defined as polymeric particles spanning nanometer- to millimeter-length scales, present persistent analytical challenges due to their wide size distributions, irregular morphologies, and heterogeneous surface chemistry [1,2,3]. These characteristics complicate reliable identification, particularly under conditions where particle geometry, surface roughness, and substrate accessibility strongly influence signal generation [4,5,6]. These challenges are further compounded by physicochemical aging processes that introduce surface roughness, oxidation, and heterogeneity, all of which can strongly influence optical coupling and spectroscopic response [4,5,6,7]. Beyond aquatic environments, microplastics have been increasingly detected in atmospheric deposition [8], urban and remote air samples [9], and precipitation [10], indicating efficient long-range transport and widespread environmental distribution. Recent studies have further reported the presence of microplastics in marine organisms [3] and food webs [11], raising growing concern regarding chronic exposure pathways and ecological impact. Although the toxicological consequences remain an active area of investigation, these observations underscore the urgency of developing reliable analytical platforms capable of identifying solid microplastic particles across diverse environmental matrices.

Raman spectroscopy is widely employed for polymer identification because of its molecular specificity and non-destructive nature [4,5]. However, conventional Raman analysis is often limited by weak scattering efficiency and fluorescence interference, especially when probing small, irregular, or aged polymer particles [5,6]. These limitations become more pronounced for heterogeneous solid particulates, where nonuniform laser-sample coupling and restricted interaction volumes reduce signal reproducibility. Consequently, reliable Raman identification of microplastics depends not only on molecular composition but also on how effectively solid particles interact with the optical probing volume and measurement substrate [4,5,6].

Surface-enhanced Raman scattering (SERS) offers a powerful approach to overcome these limitations by amplifying Raman signals through localized electromagnetic field enhancement generated by plasmonic nanostructures [12,13]. SERS has been widely explored for chemical sensing and environmental analysis due to its high sensitivity and capability for molecular fingerprint identification [13]. Recent studies have also explored hybrid Raman sensing strategies, including platforms that combine SERS with electronic detection approaches such as field-effect transistors to enhance signal transduction for complex analytes [14]. Nevertheless, for solid particulate analytes such as microplastics, SERS performance is governed not only by intrinsic plasmonic activity but also by effective particle–substrate contact and accessibility to localized electromagnetic hotspots. Reliability evaluations of commercial SERS substrates have shown that planar architectures optimized for molecular adsorption often exhibit limited hotspot accessibility and poor reproducibility when applied to three-dimensional solid particles [15,16,17]. As a result, SERS-based detection of heterogeneous particulates requires substrate architectures that can accommodate irregular particle morphologies while maintaining accessible plasmonic junctions.

Three-dimensional porous or fibrous architectures provide high surface area, interconnected junctions, and mechanically compliant networks capable of accommodating irregular solid particulates. Electrospun carbon fiber (ECF) networks, in particular, enable conformal contact with solid particles while maintaining electrical conductivity and structural robustness [18]. Recent studies on three-dimensional SERS substrates for microplastic detection demonstrate that enhancement efficiency is strongly influenced by particle size and contact geometry, highlighting the need for architectures that provide accessible plasmonic junctions across relevant length scales [19,20]. These findings suggest that substrate geometry plays a critical role in defining the practical operating window for SERS-based microplastic detection.

Microwave-assisted synthesis has emerged as an efficient approach for nanoparticle formation at conductive carbon interfaces due to its ability to generate rapid and localized heating with accelerated reaction kinetics [21,22,23]. Microwave-induced carbothermal shock processes, characterized by transient extreme thermal gradients at carbon surfaces, enable ultrafast nucleation and anchoring of metal nanoparticles without chemical reducing agents [23]. However, most reported microwave-assisted approaches rely on aqueous systems, stepwise precursor loading, or post-impregnation strategies, limiting uniform nanoparticle anchoring on three-dimensional fibrous substrates [22,23,24,25].

Importantly, for three-dimensional SERS substrates intended for solid particulate analytes, plasmonic activity alone is insufficient to ensure reliable performance. Nanoparticle size, distribution uniformity, and junction accessibility must be carefully controlled to balance electromagnetic enhancement with reproducible particle–substrate contact [26]. In this context, precursor concentration and growth kinetics play critical roles in defining nanoparticle morphology and hotspot distribution, motivating systematic evaluation of nanoparticle growth behavior prior to sensing performance assessment. Establishing an effective operating window for SERS detection of polymer particulates spanning the sub-micrometer to a tens-of-micrometers size regime under contact-limited conditions, therefore, remains an open challenge.

Here, we report a rapid microwave-assisted carbothermal shock strategy to deposit silver nanoparticles onto electrospun carbon fibers using a mixed ethanol–hexane solvent system. Localized microwave-induced heating enables uniform Ag nanoparticle nucleation and anchoring within 45 s, producing a junction-rich three-dimensional plasmonic architecture with strong nanoparticle adhesion. Unlike conventional SERS substrates optimized for molecular adsorption, the Ag nanoparticle-decorated ECF platform is designed to provide mechanically accessible plasmonic hotspots for heterogeneous solid particulates. The resulting composite substrate is evaluated using morphologically aged-like polystyrene (PS) and polyethylene terephthalate (PET) microplastics together with size-controlled PS bead standards spanning ~50 nm to 45 µm. By integrating structural characterization, Raman mapping, and finite-difference time-domain simulations, this study elucidates how particle–substrate contact geometry and near-field accessibility govern SERS response for solid polymer analytes. These results establish practical design principles for three-dimensional SERS substrates capable of probing heterogeneous microplastic particles under realistic measurement conditions.

## 2. Materials and Methods

### 2.1. Materials

Polyacrylonitrile (PAN, M.W. 150,000), N, N-dimethylformamide (DMF, 99.8%), silver (I) nitrate (AgNO_3_, 99%), n-hexane (≥99%), ethanol (200 proof), polystyrene (PS, M.W. 200,000), and polyethylene terephthalate (PET) were obtained from Sigma-Aldrich (St. Louis, MO, USA) and used as received. Polystyrene size-standard beads with nominal diameters of 0.05, 0.5, 1, and 45 µm (coefficient of variation: 2–10%; 2.5 wt% aqueous suspensions) were purchased from Polysciences (Warrington, PA, USA).

### 2.2. Methods

#### 2.2.1. Preparation of Electrospun Carbon Fiber (ECF) Substrates

ECF mats were prepared from PAN precursor fibers following established stabilization and carbonization protocols, as detailed in the Electronic Supporting Information. The resulting ECF mats exhibit continuous fibrous networks with uniform diameters and sufficient electrical conductivity for microwave-assisted processing.

#### 2.2.2. Microwave-Assisted Carbothermal Shock Synthesis of AgNPs@ECF

To deposit silver nanoparticles onto the electrospun carbon fiber (ECF) substrate, a 0.5 × 0.5 cm section of the ECF mat was immersed in a mixed-solvent system consisting of an ethanolic AgNO_3_ precursor solution and n-hexane. Specifically, 20 µL of AgNO_3_ solution (1–10 mM in ethanol) was combined with 200 µL of n-hexane, and the ECF substrate was placed directly into the mixed solvent to ensure intimate contact with both phases.

Microwave irradiation was applied using a commercial domestic microwave oven operating at 2.45 GHz with a nominal output power of 1200 W. Samples were irradiated at full power for 45 s in an open glass vial positioned at the center of the microwave cavity, without stirring or rotation. This irradiation time was selected based on the solvent evaporation behavior and substrate response under the applied microwave conditions. Under these conditions, complete solvent removal and visible incandescence of the conductive ECF network typically occurred within ~45–50 s. Shorter irradiation times often resulted in incomplete solvent removal and less uniform nanoparticle deposition, whereas longer irradiation introduces additional thermal input that may promote nanoparticle coarsening or increased surface oxidation. During irradiation, the ECF network exhibited visible incandescence, consistent with strong microwave coupling and localized Joule heating of the conductive fiber network. Simultaneously, the surrounding mixed solvent underwent vigorous boiling, indicating rapid volumetric heating of the liquid phase. Under these conditions, microwave energy is preferentially absorbed by the conductive ECF substrate rather than the surrounding low-microwave-absorption solvent, resulting in transient localized heating at the fiber surface that promotes rapid Ag nanoparticle nucleation and anchoring.

Following microwave treatment, the AgNP-functionalized ECF mats were thoroughly rinsed with ethanol to remove residual precursor species and dried under ambient conditions. Samples prepared using AgNO_3_ concentrations of 1, 3, 5, 7, and 10 mM were denoted as AgNPs@ECF-1, AgNPs@ECF-3, AgNPs@ECF-5, AgNPs@ECF-7, and AgNPs@ECF-10, respectively. This mixed ethanol–hexane microwave strategy differs from previously reported stepwise or sequential precursor-loading approaches by ensuring homogeneous precursor availability during microwave-induced carbothermal shock, thereby promoting uniform nanoparticle nucleation across the three-dimensional fibrous substrate.

#### 2.2.3. Preparation of Aged-like Microplastic Samples

PS microplastics were prepared using an anti-solvent precipitation method to introduce surface heterogeneity representative of aged environmental particles. Briefly, approximately 1 g of finely ground PS was dissolved in 100 mL of acetone under agitation. After complete dissolution, 10 mL of distilled water was added as an anti-solvent to induce polymer precipitation. The resulting precipitate was collected by vacuum filtration using a 0.45 µm membrane filter and dried at 105 °C under vacuum.

PET microplastics were prepared using a similar dissolution–reprecipitation approach with a different solvent system. Ground PET powder was dissolved in concentrated trifluoroacetic acid (TFA) and subsequently reprecipitated by dilution with aqueous TFA (10 vol%), inducing polymer precipitation. The precipitated PET was collected and dried following the same procedure as PS.

#### 2.2.4. Microplastic Loading onto AgNPs@ECF Substrates

For SERS measurements, microplastic particles were deposited onto the AgNPs@ECF substrates using a vacuum-assisted filtration approach to ensure reproducible particle–substrate contact. The AgNPs@ECF substrate was placed on top of a pre-wetted 0.2 µm nylon membrane filter, and 100 µL of the microplastic suspension was pipetted onto the substrate under gentle vacuum. After filtration, samples were air-dried for at least 30 min before Raman measurements.

#### 2.2.5. Structural and Chemical Characterization

The morphology of ECF and AgNPs@ECF substrates was characterized using scanning electron microscopy (SEM; Gemini Ultra 55, Zeiss). Fiber diameters and Ag nanoparticle size distributions were analyzed using ImageJ software version 2.0. Nanoparticle size distributions were evaluated using cumulative particle fraction analysis, from which characteristic diameters (d10, d50, and d90) and the distribution span were extracted to assess distribution uniformity.

Energy-dispersive X-ray spectroscopy (EDS) was performed using a Phenom ProX benchtop scanning electron microscope (Thermo Scientific, Waltham, MA, USA). X-ray diffraction (XRD) patterns were collected using a Panalytical Empyrean diffractometer equipped with a Cu Kα radiation source operating at 45 kV and 40 mA. X-ray photoelectron spectroscopy (XPS) measurements were conducted on a Thermo Scientific ESCALAB 250Xi system with an Al Kα source, with charge compensation applied and binding energies referenced to the C 1s peak at 284.8 eV. High-resolution transmission electron microscopy (HRTEM) and high-angle annular dark-field scanning transmission electron microscopy (HAADF-STEM), together with STEM–EDS elemental mapping, were performed using an FEI Tecnai F30 microscope operated at an accelerating voltage of 300 keV.

#### 2.2.6. Raman and SERS Measurements

Raman spectroscopy was performed using a Horiba LabRAM Evolution micro-Raman spectrometer equipped with a 532 nm Nd:YAG laser and a 600 lines mm^−1^ diffraction grating. The excitation wavelength was selected to maximize plasmonic coupling with the AgNP-decorated substrate. Spectra were acquired using an integration time of 10 s and averaged over two accumulations, with laser power maintained at 0.63 mW for all measurements.

Measurements were performed using both 10× (N.A. = 0.25) and 50× (N.A. = 0.5) objectives to probe SERS performance under different optical sampling conditions relevant to laboratory-based and portable Raman systems.

SERS signal enhancement (SE) was calculated using:

$${SERS}_{SE}=\frac{I_{SERS}}{I_{non-SERS}}$$

where $I_{SERS}$ is the Raman intensity measured on AgNPs@ECF-5 and $I_{non-SERS}$ is the corresponding intensity measured on bare ECF substrates. Because Raman spectra from heterogeneous microplastic particles can exhibit an intrinsic fluorescence background, SE values are reported as a semi-quantitative metric under practical measurement conditions. Intensities were extracted consistently using identical acquisition parameters for both AgNPs@ECF and bare ECF control measurements, and reproducibility statistics are provided in Figures S7–S9.

#### 2.2.7. Finite-Difference Time-Domain (FDTD) Simulations

Electromagnetic simulations were performed using ANSYS Lumerical FDTD Solutions to qualitatively examine near-field enhancement and plasmonic coupling behavior in Ag nanoparticle-decorated electrospun carbon fiber architectures. The simulated geometry consisted of a single cylindrical carbon fiber (radius ≈ 0.2 µm) decorated with spherical Ag nanoparticles (radii ≈ 20–30 nm) partially embedded on the fiber surface, consistent with experimentally observed morphologies. Optical constants for silver and carbon were taken from the built-in “Ag-Palik” and “Carbon-Querry 1985” material datasets, respectively. Perfectly matched layer (PML) boundary conditions were applied in all directions, and a broadband plane-wave source (400–800 nm) was incident laterally with polarization perpendicular to the fiber axis. Electric field intensity distributions (|E|^2^) were extracted at 532 nm to correspond with experimental excitation conditions. These simulations are intended to provide qualitative insight into localized field confinement at AgNP–fiber junctions and rapid near-field decay away from the substrate surface, rather than quantitative prediction of absolute enhancement factors. Because the experimentally observed nanoparticles exhibited irregular shapes and surface defects, simplified geometries were used to capture the dominant electromagnetic behavior rather than reproduce the exact experimental morphology.

## 3. Results and Discussion

### 3.1. Synthesis of AgNPs@ECF via Microwave Irradiation

**Figure 1:**
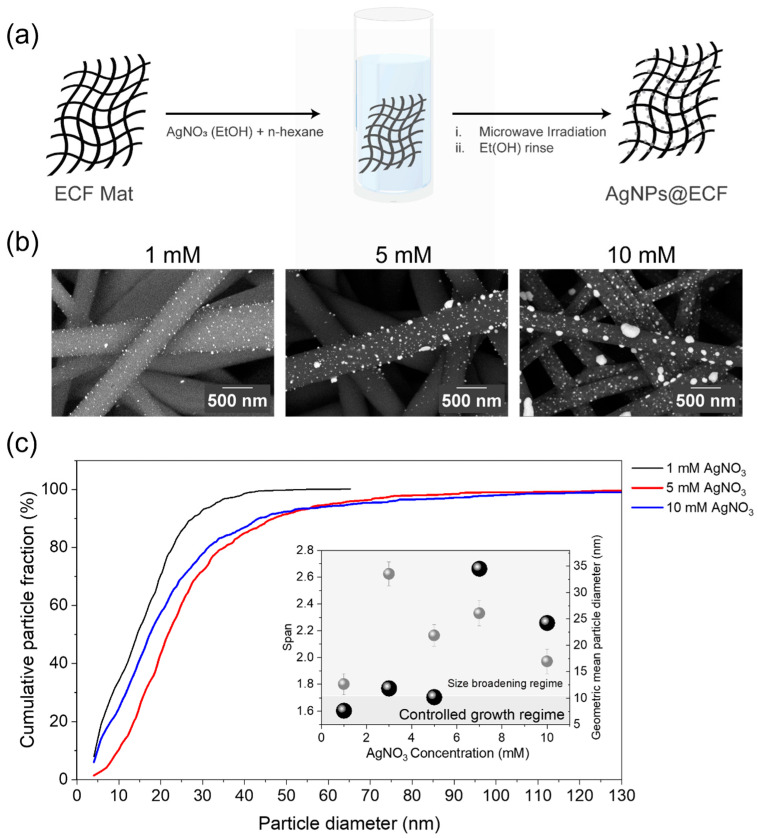
(**a**) Schematic of the microwave-assisted carbothermal shock synthesis of Ag nanoparticles on ECF mats using a mixed ethanolic AgNO_3_/n-hexane system. (**b**) Backscattered SEM images of AgNPs@ECF prepared with AgNO_3_ concentrations of 1, 5, and 10 mM (scale bars: 500 nm). (**c**) Cumulative number-based particle size distributions (CDFs) of Ag nanoparticles formed at different precursor concentrations. Inset: Geometric mean particle diameter and PSD span versus AgNO_3_ concentration, indicating a transition from controlled growth to size broadening at higher concentrations.

The AgNPs@ECF composite was synthesized via a microwave-assisted carbothermal shock method, as schematically illustrated in Figure 1a. Owing to its high vapor pressure and negligible microwave absorption, hexane primarily acts as a surrounding medium during microwave irradiation, while energy absorption occurs preferentially within the conductive ECF network. As a result, localized Joule heating is concentrated at the carbon fibers, producing steep thermal gradients at the fiber–solvent interface. This response is consistent with the turbostratic carbonaceous nature of the ECF substrate, with structural defects identified in the Raman spectrum (Figure S1c) potentially enhancing microwave coupling [21]. The observed incandescence of the conductive ECF network under microwave irradiation is consistent with prior reports demonstrating preferential microwave absorption and rapid localized heating in conductive carbon frameworks, which can drive ultrafast reduction and nucleation of metal species at carbon interfaces [22,23,24]. In particular, microwave-induced carbothermal shock has been shown to generate transient high-temperature interfacial environments capable of enabling rapid surface functionalization and nanoparticle anchoring without added chemical reductants [23].

The resulting transient high-temperature interfacial environment is consistent with accelerated Ag^+^ reduction and promotes rapid nanoparticle nucleation directly at the fiber surface without the need for chemical reducing agents. Consequently, Ag nanoparticles are preferentially anchored along the ECF network, yielding a mechanically robust, three-dimensional plasmonic composite. Within this framework, the present ethanol–hexane configuration maintains precursor availability while minimizing bulk microwave absorption by the surrounding liquid phase, thereby concentrating thermal energy at the fiber–solution interface where nucleation and growth occur [21,23].

To ensure uniform precursor dispersion and suppress phase separation between AgNO_3_ and hexane, ethanol was employed as the precursor solvent rather than water. Unlike water, which is immiscible with hexane, ethanol exhibits amphiphilic behavior that stabilizes the mixed-solvent system and enables homogeneous precursor delivery to the fiber surface. This solvent configuration supports controlled AgNP nucleation under microwave irradiation, as the ECF selectively absorbs microwave energy and concentrates thermal energy at the fiber–solution interface. The uniform Ag nanoparticle distribution observed across the electrospun carbon fiber network highlights the advantage of this mixed-solvent microwave approach, in which precursor availability and localized heating occur concurrently throughout the three-dimensional architecture rather than being limited by sequential diffusion or surface wetting.

AgNO_3_ concentrations ranging from 1 to 10 mM were systematically investigated to evaluate their influence on nanoparticle growth behavior. Concentrations exceeding 10 mM resulted in macroscopic phase separation of the precursor solution from hexane and were therefore excluded. Backscattered SEM images (Figure 1b) reveal dense decoration of AgNPs along the ECF surface across all investigated concentrations, with increasing precursor concentration leading to progressively larger particle sizes and broader size distributions. Additional SEM images are provided in Figure S2. SEM–EDS analysis confirmed the presence of Ag and revealed a near-linear relationship between AgNO_3_ concentration and Ag loading (Figure S3, R^2^ = 0.98), indicating that silver deposition within this concentration range is primarily governed by precursor availability.

To quantitatively assess nanoparticle growth and distribution uniformity, particle size distribution (PSD) analysis was performed using individual particle diameters extracted from SEM images [27]. The resulting number-based PSDs, shown in Figure 1c, exhibit right-skewed profiles consistent with log-normal behavior. Characteristic percentile diameters (d10, d50, and d90) were extracted to describe distribution breadth, while the geometric mean diameter and PSD span were used as primary descriptors of growth behavior and size uniformity. The corresponding numerical values for all PSD metrics are summarized in Table S1.

As summarized in the inset of Figure 1c, increasing AgNO_3_ concentration initially promotes controlled particle growth, reflected by a gradual increase in geometric mean diameter accompanied by relatively low span values within the 1–5 mM concentration range. This regime is characterized by modest growth with limited broadening, consistent with a growth-dominated nucleation process. At higher precursor concentrations (≥7 mM), a pronounced increase in span is observed together with a non-monotonic evolution of the geometric mean diameter, indicating a transition to a size-broadening regime. This behavior is consistent with a broader growth regime that may involve concurrent nucleation and aggregation under conditions of elevated precursor availability. However, time-resolved kinetic analysis was not performed in this study, and therefore, the mechanistic interpretation should be considered as a qualitative assumption. Notably, although higher precursor concentrations increase overall Ag loading, the geometric mean particle diameter does not increase monotonically, and at 10 mM AgNO_3_, a reduced mean diameter was observed alongside substantially broadened distributions. This trend suggests that growth is not governed solely by particle coarsening at higher precursor concentrations and may involve competing nucleation and growth processes, consistent with nanoparticle synthesis literature, although this interpretation remains qualitative in the absence of time-resolved kinetic measurements.

Among the investigated conditions, AgNPs@ECF-5 exhibits an optimal balance between particle growth and size uniformity, combining a moderate geometric mean diameter with the lowest span among the higher-concentration samples. While surface coverage was qualitatively assessed from SEM images, quantitative determination of coverage on the curved, three-dimensional fibrous network remains inherently ambiguous. Accordingly, PSD-derived metrics, including percentile diameters, geometric mean diameter, and span, were employed to evaluate nanoparticle growth control and morphological uniformity relevant to plasmonic hotspot accessibility.

**Figure 2:**
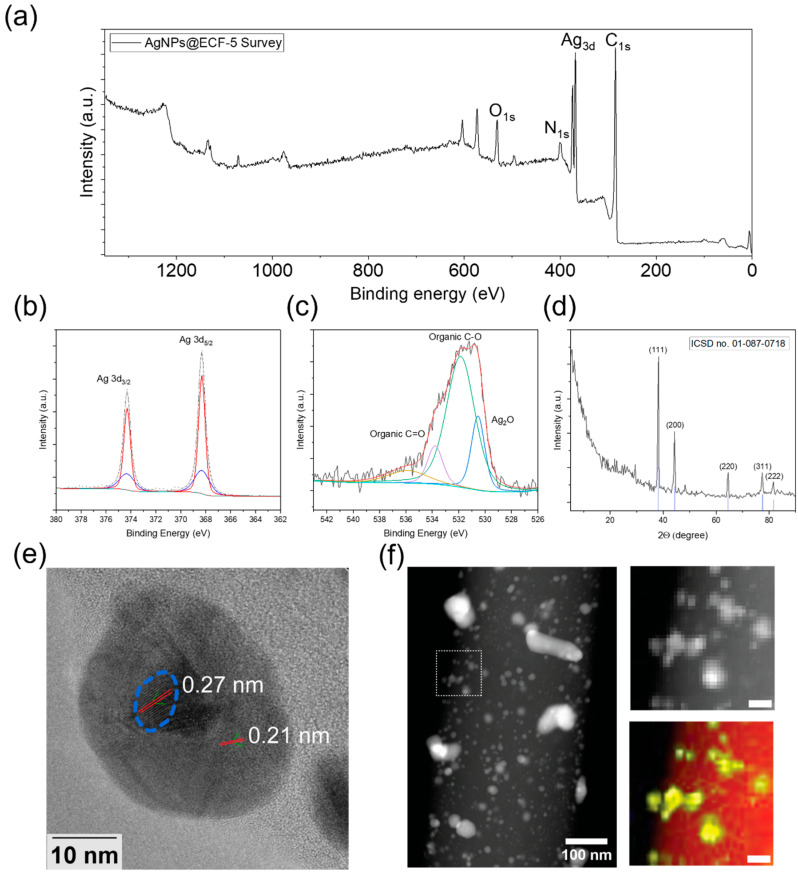
Structural and chemical characterization of AgNPs@ECF-5. (**a**) XPS survey spectrum confirming the presence of Ag, C, N, and O. (**b**) High-resolution Ag 3d XPS spectrum showing dominant metallic Ag with minor oxidized contributions. (**c**) O 1s XPS spectrum deconvoluted into Ag_x_O and oxygen-containing functional groups. (**d**) XRD pattern showing diffraction peaks of face-centered cubic Ag (ICSD No. 01-087-0718). (**e**) HRTEM image of a Ag nanoparticle on the ECF surface, with lattice spacings corresponding to Ag(111) and Ag_2_O(111). (**f**) HAADF-STEM image and corresponding STEM–EDS elemental maps of the selected region confirming localized Ag nanoparticle deposition on the fibrous substrate.

Based on the controlled growth behavior and favorable distribution uniformity, AgNPs@ECF-5 was selected as a representative substrate for detailed structural and chemical characterization. XPS survey spectra (Figure 2a) confirm the presence of Ag, C, N, and O. High-resolution Ag 3d spectra (Figure 2b) show dominant metallic Ag contributions at 368.2 eV (Ag 3d_5_/_2_) and 374.2 eV (Ag 3d_3_/_2_), with minor components attributable to Ag_2_O. The corresponding O 1s spectrum (Figure 2c) indicates contributions from Ag_x_O species as well as C–O and C=O environments. XRD patterns (Figure 2d) further confirm the formation of face-centered cubic Ag, with diffraction peaks indexed to the Ag(111), Ag(200), Ag(220), Ag(311), and Ag(222) planes. The high-resolution C 1s spectrum (Figure S4) is dominated by contributions from the ECF substrate, with minor oxygen-containing carbon functionalities consistent with limited surface oxidation.

HRTEM images (Figure 2e) reveal AgNPs localized along the ECF surface, with lattice fringes corresponding to Ag(111) (~0.21 nm) [25] and minor Ag_2_O(111) (~0.28 nm) [28] domains. The preferential localization of AgNPs at the fiber surface supports nucleation and growth occurring predominantly at the fiber–solution interface. Minor surface oxidation observed by XPS and HRTEM is likely introduced during post-synthesis air exposure or under mixed-solvent reaction conditions. HAADF-STEM images and the corresponding STEM–EDS elemental maps are shown in Figure 2f. The overlaid C and Ag maps indicate that the majority of the nanoparticles consist of metallic Ag, with minor O contributions observed (Figure S5), consistent with the HRTEM observations.

Compared with conventional SERS substrate fabrication approaches, this microwave-assisted method is rapid, scalable, and reagent-efficient. Compared with conventional SERS substrate fabrication routes that rely on chemical reductants, multistep impregnation, or vacuum and lithographic processing [17,22,23,24], the present microwave-assisted carbothermal shock approach achieves Ag nanoparticle formation and anchoring on a three-dimensional fibrous carbon network within 45 s while preserving a junction-rich architecture tailored for contact-limited particulate sensing [23].

The resulting AgNPs@ECF composite forms a junction-rich, three-dimensional plasmonic architecture with mechanically compliant and analyte-accessible interfaces. These features provide a structural foundation for effective particle–substrate contact and reproducible SERS performance, motivating the subsequent evaluation of AgNPs@ECF-5 using representative polymer standards and aged microplastic analytes.

### 3.2. SERS of Aged Microplastics on AgNPs@ECF-5

To better reflect the heterogeneous surface characteristics of environmentally relevant microplastics, PS and PET samples were prepared via partial dissolution in a polymer-specific solvent (acetone for PS and TFA for PET), followed by precipitation. This treatment introduced surface irregularities and morphological heterogeneity while preserving the polymer backbone, yielding microplastic particles with non-idealized surfaces that approximate selected morphological features of environmentally weathered particles. The term aged-like is used here to denote laboratory-treated microplastics designed to reproduce selected surface features associated with environmental aging, rather than to imply full equivalence to natural aging processes [7]. The anti-solvent precipitation approach primarily introduces morphological heterogeneity and surface roughness that mimic certain physical aspects of environmental fragmentation. However, environmental weathering can also involve UV exposure, oxidative degradation, and mechanical abrasion that introduce chemical surface modifications (e.g., carbonyl or hydroxyl groups). Spectroscopic characterization of chemical aging (e.g., FTIR or XPS analysis of the polymers) was not performed in this study; therefore, the term “aged-like” refers to morphology-based physical aging rather than confirmed chemical oxidation. Accordingly, the SERS performance evaluated in this study reflects realistic solid particle–substrate interactions rather than idealized flat polymer surfaces.

Using these representative microplastic models, the SERS performance of the AgNPs@ECF-5 composite was evaluated by examining its ability to enhance Raman signals from PS and PET. To verify the intrinsic plasmonic activity of the substrate, 4-mercaptobenzoic acid (4-MBA) was first employed as a molecular probe (Figure S6). Owing to the strong affinity of the thiol (–SH) group for Ag surfaces, an enhancement factor (EF) of 2.4 × 10^5^ was obtained at 1582 cm^−1^, corresponding to the aromatic ring-breathing mode. This value confirms the intrinsic SERS activity of the AgNPs@ECF-5 substrate and is provided primarily as a reference to demonstrate plasmonic activity rather than to benchmark performance against planar molecular-adsorption-based SERS substrates.

In contrast, microplastics are heterogeneous solid particles for which the number of molecules contributing to the Raman signal cannot be reliably determined due to variable particle–substrate contact geometry and sampling depth. Therefore, enhancement factors were not adopted as a quantitative metric for subsequent experiments. Instead, relative signal enhancement was used as a semi-quantitative metric to evaluate microplastic detection performance.

**Figure 3:**
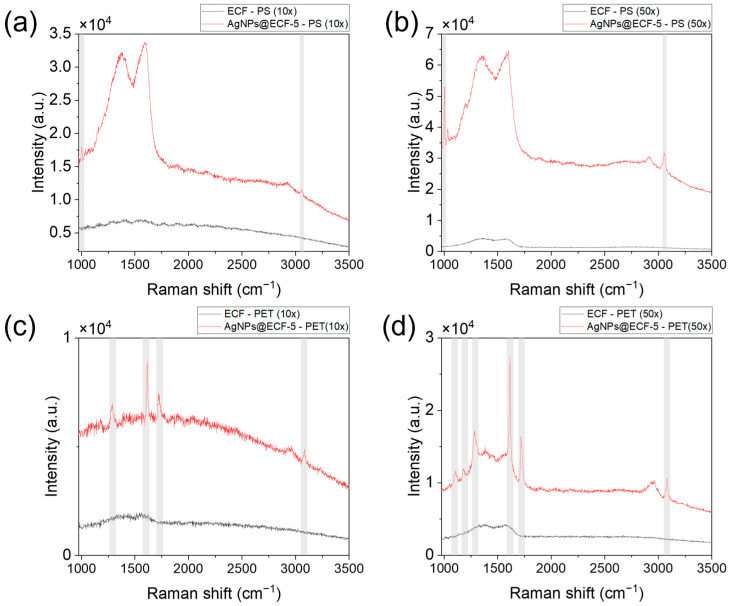
Representative Raman spectra of PS and PET microplastics measured on bare ECF (black) and AgNPs@ECF-5 (red) under identical acquisition conditions. (**a**) PS, 10×. (**b**) PS, 50×. (**c**) PET, 10×. (**d**) PET, 50×. Shaded regions denote characteristic polymer bands used for SE analysis. Spectra are shown without baseline subtraction; intensity scales differ between panels.

The SE values were calculated based on direct comparison of raw Raman intensities measured on AgNPs@ECF-5 and bare ECF substrates under identical conditions, providing a practical assessment of SERS performance for solid particulate analytes. Raman spectra were collected using both 10× and 50× objective lenses to probe enhancement behavior under different optical configurations. Representative spectra are shown in Figure 3, and the corresponding Raman peak assignments and SE values are summarized in Table 1.

Using the 10× objective, the AgNPs@ECF-5 substrate exhibited SE values exceeding 2.5 for both PS and PET (Figure 3a,c), with PS showing slightly higher enhancement, which may reflect differences in Raman scattering cross section and particle-substrate contact configuration under the present measurement conditions. When measured with the 50× objective (Figure 3b), PS exhibited substantially higher enhancement, with SE values exceeding 20 at several characteristic Raman bands. The strongest enhancement was observed at 1000.9 cm^−1^, followed by peaks at 3054.4 cm^−1^ and 1034 cm^−1^. In contrast, PET (Figure 3d) displayed moderate enhancement across multiple vibrational modes, with SE values of 4.29 at 1108 cm^−1^, 4.07 at 1180 cm^−1^, 4.48 at 1285 cm^−1^, 6.84 at 1615 cm^−1^, and 6.43 at 1720 cm^−1^. The comparatively lower enhancement observed for PET may be influenced by differences in Raman scattering cross section, particle morphology, and particle–substrate contact geometry, which affect local electromagnetic coupling within the fibrous substrate.

The influence of optical configuration on SERS performance was further assessed by comparing measurements obtained using 10× and 50× objectives. The higher numerical aperture of the 50× objective enables enhanced spatial resolution and more effective sampling of localized plasmonic hotspots, thereby revealing the intrinsic enhancement capability of the AgNPs@ECF-5 substrate. In contrast, the 10× objective approximates measurement conditions relevant to portable Raman systems with lower laser power density and spatial resolution. Importantly, consistent enhancement trends were observed across both objectives, underscoring the robustness of the AgNPs@ECF-5 composite under varying measurement conditions.

To quantitatively evaluate signal uniformity and spot-to-spot reproducibility under realistic solid-particle measurement conditions, Raman spectra were collected from 16 randomly selected locations on PS-spiked AgNPs@ECF-5 substrates using identical acquisition parameters. Raw three-dimensional waterfall spectra (Figure S7) display consistent absolute intensities and preserved spectral features across all sampling locations, indicating the absence of single-hotspot-dominated behavior. For clarity of spectral comparison, the corresponding normalized Raman spectra are shown in Figure S8, highlighting the consistent presence and relative intensity hierarchy of characteristic PS vibrational modes across all measurement locations. Statistical analysis of the raw (non-normalized) Raman intensities at characteristic PS bands (1000, 1600, and 3055 cm^−1^) yields relative standard deviations on the order of ~50–60% (Figure S9). These RSD values reflect variations in particle size, orientation, and local contact geometry inherent to solid microplastics interacting with a three-dimensional fibrous substrate, rather than instability of the AgNPs@ECF platform. Despite this variability, all measurement locations yield comparable absolute signal levels and preserved spectral signatures, confirming reproducible detection under practical, contact-limited solid-particle SERS conditions. Comparable variability has been reported for solid-particle SERS measurements on three-dimensional substrates, where particle placement and contact geometry dominate signal fluctuations.

SEM imaging of microplastic particles deposited on the AgNPs@ECF substrate (Figure S10) reveals direct physical contact between polymer particles and multiple fiber segments within the three-dimensional network. In contrast to planar substrates, the interconnected fibrous architecture enabled microplastic particles to simultaneously engage several AgNP-decorated fiber junctions, depending on particle size and orientation. This contact configuration supported efficient near-field coupling between plasmonic hotspots and the solid polymer surface and was consistent with the contact-limited enhancement behavior observed in the SERS measurements.

Hyperspectral Raman mapping was performed to further examine the spatial enhancement behavior. Figure S11a,b display the Raman intensity distribution of PS at 1000.9 cm^−1^, together with an overlay of the Raman map and the corresponding optical image. Enhanced Raman signals are predominantly localized near particle edges. This spatial variation is likely associated with differences in particle–substrate contact geometry and near-field accessibility within the fibrous AgNPs@ECF architecture. In particular, particle edges may provide more effective mechanical contact with multiple AgNP-decorated fiber junctions, promoting stronger local electromagnetic coupling.

Comparable mapping of PET microplastics (Figure S11c,d) shows a more uniform intensity distribution across the particle surface, with enhanced regions frequently observed near particle centers for particles ranging from approximately 1 to 18 μm. These differences likely reflect variations in particle morphology, local contact configuration, and hotspot accessibility rather than intrinsic differences in polymer chemistry alone.

### 3.3. Size-Dependent SERS Response and Contact Effects

To further probe the role of particle–substrate contact geometry, size-dependent SERS behavior was investigated using PS bead standards with well-defined diameters spanning the nanometer to micrometer regime. Representative Raman spectra acquired on AgNPs@ECF-5 show clear enhancement of characteristic PS vibrational modes relative to the ECF control for particle sizes of 50 nm and 0.5 μm, whereas only marginal enhancement is observed for larger particles (45 μm). Signal enhancement was quantified using the PS ring-breathing mode at approximately 1000 cm^−1^, which is well separated from the carbon D and G bands and remains detectable across all particle sizes.

The size-dependent SERS response observed here is broadly consistent with emerging reports on SERS detection of solid micro- and nanoplastic particles, which demonstrate that signal intensity varies nonlinearly with particle size due to differences in analyte–substrate coupling and hotspot accessibility rather than polymer chemistry alone [19,20]. Recent studies employing three-dimensional plasmonic architectures, including hierarchically porous Ag foams, have shown that pretreatment-free SERS detection of microplastics is strongly influenced by substrate morphology and analyte size, with enhancement governed by near-field accessibility rather than bulk material properties [19].

**Figure 4:**
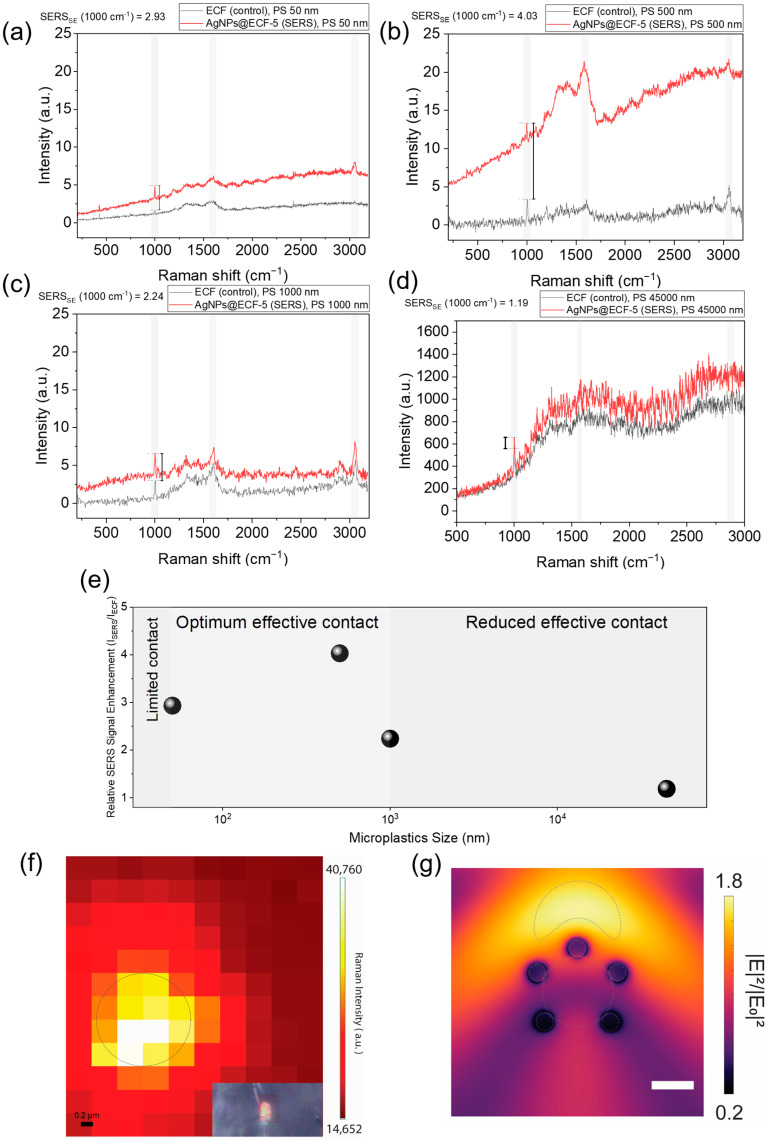
Size- and contact-dependent SERS response of PS on AgNPs@ECF-5. (**a**–**d**) Raman spectra of PS on bare ECF (black) and AgNPs@ECF-5 (red) under identical conditions, highlighting enhancement of the PS band near 1000 cm^−1^ for different particle sizes (no baseline subtraction). Shaded regions indicate bands used for SE analysis. (**e**) Relative SERS enhancement versus PS particle size, showing a non-monotonic dependence with maximum enhancement in the sub-micrometer regime. (**f**) Raman intensity map of a 0.5 µm PS particle at 1000.9 cm^−1^ acquired using EM gain, with an enhancement hotspot observed in the selected region (qualitative). (**g**) FDTD-simulated electric field intensity distribution (|E|^2^/|E_0_|^2^) around AgNP clusters on a carbon fiber, with hotspot located on the selected region, illustrating localized field confinement and near-field decay.

Figure 4a–d show the SERS Raman spectra obtained from PS particles with sizes of 50 nm (Figure 4a), 0.5 µm (Figure 4b), 1 µm (Figure 4c), and 45 µm (Figure 4d). All spectra exhibited Raman signal enhancement, as evaluated from the intensity differences at ~1000 cm^−1^, corresponding to the PS ring-breathing mode. The highest enhancement was observed for the 0.5 µm particles, whereas the lowest enhancement was observed for the 45 µm particles.

As summarized in Figure 4e, the relative SERS enhancement exhibited a non-monotonic dependence on particle size. For the smallest particles (50 nm), the enhancement was limited, which is attributed to a reduced effective contact area and incomplete overlap between the particle volume and the plasmonic hotspots distributed along the fibrous substrate. Increasing the particle size to the sub-micrometer regime (0.5 µm) resulted in a pronounced increase in enhancement, consistent with an optimal contact condition in which the particle can simultaneously engage multiple AgNP-decorated fiber junctions. In contrast, further increases in particle size led to a progressive reduction in enhancement. For larger microplastics (≥1 µm), only a small fraction of the particle surface resides within the near-field decay length of the plasmonic hotspots, resulting in diminished electromagnetic coupling despite the larger particle volume.

Similar reductions in enhancement for both nanoplastics (≥50 nm) and large microplastics (<45 µm) have been reported in prior studies and are commonly attributed to limited effective contact area and the rapid decay of localized electromagnetic fields away from plasmonic junctions [20]. In this context, analyte size imposes a geometric constraint on near-field overlap, thereby defining a practical operating window for solid-particle SERS detection.

The comparatively lower enhancement observed for smooth PS bead standards relative to aged PS microplastics highlighted the dominant role of particle–substrate contact geometry in governing the SERS response. Aged microplastics exhibited irregular morphologies and increased surface roughness, enabling multiple contact points and greater overlap with the plasmonic near field of the AgNP-decorated fibrous substrate. In contrast, spherical PS beads primarily engaged the substrate through limited point contact, restricting effective near-field coupling.

To further elucidate the origin of the non-monotonic size dependence, spatially resolved Raman mapping was performed on a 0.5 µm PS particle (Figure 4f). The resulting map revealed highly localized regions of enhanced intensity concentrated at the particle–fiber interface, indicating that SERS enhancement is governed primarily by localized contact with plasmonic hotspots rather than by the bulk particle volume. The mapping was acquired using EM gain to emphasize spatial intensity variations and was therefore not used for quantitative comparison.

Complementary FDTD simulations (Figure 4g) supported this interpretation by demonstrating strong electric field confinement in the vicinity of AgNP clusters, along with a rapid near-field decay away from the substrate surface in the cross-sectional view. These simulations were used to qualitatively illustrate trends in near-field localization and decay and are not intended to predict absolute SERS enhancement values.

It should be noted that the size-dependent analysis presented here was intended to establish qualitative, contact-limited trends rather than absolute enhancement thresholds. This reflected the inherent experimental challenges associated with achieving uniform optical excitation and reproducible mechanical coupling for solid polymer particulates across a broad size range.

Collectively, these results demonstrate that effective particle-substrate contact and near-field accessibility, rather than particle size or polymer identity alone, govern SERS performance for solid microplastic analytes on three-dimensional plasmonic substrates. While previous studies have reported size-dependent SERS responses for microplastic particles, these observations are often described empirically without explicitly linking enhancement behavior to analyte–substrate contact configuration. In contrast, the present study combines experimental Raman mapping and electromagnetic simulations to provide insight into how particle–substrate contact geometry within a three-dimensional fibrous architecture influences localized plasmonic coupling and SERS enhancement [17,19,20].

These findings highlight the importance of mechanically accessible plasmonic hotspots for heterogeneous solid particulates, where analyte–substrate contact and near-field overlap define the effective detection regime. In this context, three-dimensional fibrous architectures provide structural advantages by enabling multiple particle–fiber contact points and improved hotspot accessibility compared with conventional planar substrates designed primarily for molecular adsorption.

## 4. Conclusions

This work demonstrates a rapid microwave-assisted carbothermal shock strategy for fabricating silver nanoparticle-decorated electrospun carbon fibers (AgNPs@ECF) as a three-dimensional SERS substrate designed for solid microplastic detection. Localized microwave-induced heating in a mixed ethanol–hexane system enables Ag nanoparticle nucleation and anchoring on conductive carbon fibers within 45 s, producing a mechanically compliant, junction-rich architecture without chemical reductants, vacuum processing, or lithographic patterning.

Using morphologically weathered (aged-like) PS and PET microplastics and size-controlled PS bead standards (~50 nm to 45 µm), the SERS response on AgNPs@ECF was found to be governed primarily by particle–substrate contact geometry and near-field accessibility rather than polymer identity alone. In particular, the strongest enhancement occurs in the sub-micrometer regime, where particles can simultaneously engage multiple AgNP-decorated fiber junctions and effectively access localized plasmonic near fields. In contrast, ultrasmall particles exhibit reduced enhancement due to limited effective contact and hotspot overlap, while large, smooth microparticles show diminished coupling because only a small fraction of the particle surface resides within the near-field decay length.

Spatially resolved Raman mapping and electromagnetic simulations provide direct experimental and qualitative theoretical evidence supporting a contact-dominated enhancement mechanism, revealing localized field confinement at particle–fiber interfaces and rapid near-field decay away from AgNP junctions. These results move beyond empirical size-dependent trends by explicitly linking SERS performance to mechanically accessible contact geometry within a three-dimensional fibrous architecture.

Rather than optimizing enhancement factors under idealized molecular adsorption conditions, this study highlights how substrate architecture and analyte-substrate contact govern SERS performance for solid particulate analytes. The results highlight the importance of mechanically compliant architectures and contact-accessible plasmonic junctions in defining a practical operating window for solid-particle SERS detection. By integrating rapid synthesis, structural robustness, and analyte-accessible hotspot formation, AgNPs@ECF provides a scalable and application-relevant platform for microplastic analysis.

From a practical standpoint, the fabrication strategy combines electrospinning-based fiber production with rapid microwave-assisted nanoparticle deposition, both of which are compatible with scalable processing without requiring vacuum systems or complex lithographic steps.

More broadly, this work provides general design guidance for engineering three-dimensional SERS interfaces capable of probing heterogeneous solid particulates under realistic, contact-limited measurement conditions, with potential relevance to environmental sensing and related solid-state analytical applications.

## Figures and Tables

**Table 1:** Raman peak assignments and signal enhancement factors for PS and PET samples on AgNPs@ECF-5.

Compounds	Raman Peak Location (cm^−1^)		Corresponded Peak	Raman Signal Enhancement	
	Objective Lens			Objective Lens	
	10×	50×		10×	50×
PS	1000.9	1000.9	Aromatic C-H bond out-of-plane bending mode	3.16	34.4
	1030.0	1034.0	Aromatic C-H bond in-plane bending mode	3.02	22.0
	3054.0	3050.0	Aromatic C-H bond stretching vibration	2.65	27.1
PET	-	1108.0	Aliphatic C-H bending	-	4.29
	1186.0	1180.0	Aromatic C-C stretching vibration	3.79	4.07
	1289.0	1285.0	Aromatic C-H bending mode	4.01	4.48
	1615.0	1615.0	Aromatic C=C stretching vibration	4.79	6.84
	1725.0	1720.0	C=O stretching vibration	4.79	6.43
	3081.0	3081.0	Aromatic C-H stretching	4.43	4.79

## Data Availability

The original contributions presented in this study are included in the article/Supplementary Material. Further inquiries can be directed to the corresponding authors.

## Supplementary Materials

The following supporting information can be downloaded at: https://www.mdpi.com/article/10.3390/ma19061074/s1, Figure S1: ECF fabrication and characterization; Figure S2: Additional SEM images of AgNPs@ECF prepared with various precursor concentration; Figure S3: Linear fit of atomic Ag concentration as a function of AgNO_3_ precursor concentration; Figure S4: High-resolution C_1s_ XPS spectrum with peak deconvolution; Figure S5; Additional STEM-EDS elemental maps with corresponding EDS spectrum; Figure S6: SERS spectra of 4-MBA on AgNPs@ECF-5; Figure S7: 3D waterfall plot of SERS spectra collected on multiple spots; Figure S8: Overlay of the normalized SERS spectra collected on multiples spots; Figure S9: SEM image of AgNPs@ECF-5 with microplastic; Figure S10: RSD of SERS intensities across multiple spot of aged PS samples; Figure S11: Hyperspectral Raman map of aged PET and PS microplastics. Table S1: Statistical PSD parameters of AgNPs on ECF prepared with AgNO_3_ precursor at different concentration.

## Abbreviations

The following abbreviations are used in this manuscript:

SERS	Surface-enhanced Raman scattering
AgNPs	Silver nanoparticles
ECF	Electrospun carbon fiber
PSD	Particle size distribution
PS	Polystyrene
PET	Polyethylene terephthalate
TFA	Trifluoroacetic acid
SEM	Scanning electron microscope
EDS	Energy-dispersive X-ray spectroscopy
HRTEM	High-resolution transmission electron microscopy
HAADF	High-angle annular dark-field scanning
FDTD	Finite-difference time domain
PML	Perfectly matched layer
4-MBA	4-mercaptobenzoic acid
