# Supplementary material for: Contact-Accessible Silver Nanoparticle-Decorated Electrospun Carbon Fibers for Microplastics Detection by SERS
Source: Materials (Basel). 2026 Mar 11;19(6):1074. doi: 10.3390/ma19061074 (PMC13027646; doi:10.3390/ma19061074)
Supplement: Supplementary file 1 [file materials-19-01074-s001.zip › materials-4182200-supplementary.pdf]

Supporting information

Electrospun Carbon Fiber Fabrication and Morphology

Figure S1a illustrates the visual transformation of the electrospun 10 wt% PAN fiber mat through stabilization and carbonization. Initially, the as-spun fibers are white and exhibit a distinct nitrile (-C≡N) functional group, as indicated by the FTIR peak at ~2245 cm^{-1} (Figure S1b).

Upon thermal stabilization at 260°C for 4 hours, the fibers transition to a brown color, marking the loss of the nitrile (-C≡N) functional group. This transformation is accompanied by the emergence of a new peak at ~1578 cm^{-1}, corresponding to the formation of a -C=N- group. This shift signifies the development of polyaromatic structures, which serve as precursor templates for turbostratic carbon fibers.

During pyrolysis at 900°C, the peak associated with the stabilized fibers broadens into a shoulder at ~1507 cm^{-1}, signifying increased structural disorder. At this stage, the fibers turn black, indicating the formation of a turbostratic carbon structure.

Raman analysis (Figure S1c) reveals two distinct peaks at ~1350 cm^{-1} (D-band) and ~1580 cm^{-1} (G-band). These peaks correspond to the disordered carbon structure (D-band) and the ordered graphitic structure (G-band), respectively. The fibers exhibit an I_D/I_G ratio of 1.49, which is characteristic of electrospun carbon fibers due to the presence of structural defects.

The morphological changes during each stage are depicted in Figures 1d–1f. Initially, the as-spun fibers have an average diameter of 1.15 ± 0.17 μm. After stabilization, the fibers shrink to 0.77 ± 0.08 μm, a reduction attributed to crosslinking as the nitrile (-C≡N) groups transform into -C=N- bonds. During carbonization, the fibers shrink further to 0.37 ± 0.03 μm, reflecting additional crosslinking and the development of the graphitic carbon structure.

These transformations confirm the formation of a turbostratic graphitic carbon structure within the electrospun carbon fibers. This structure is essential for enabling the ECF

substrate to effectively absorb microwaves, facilitating rapid reduction through Joule heating.

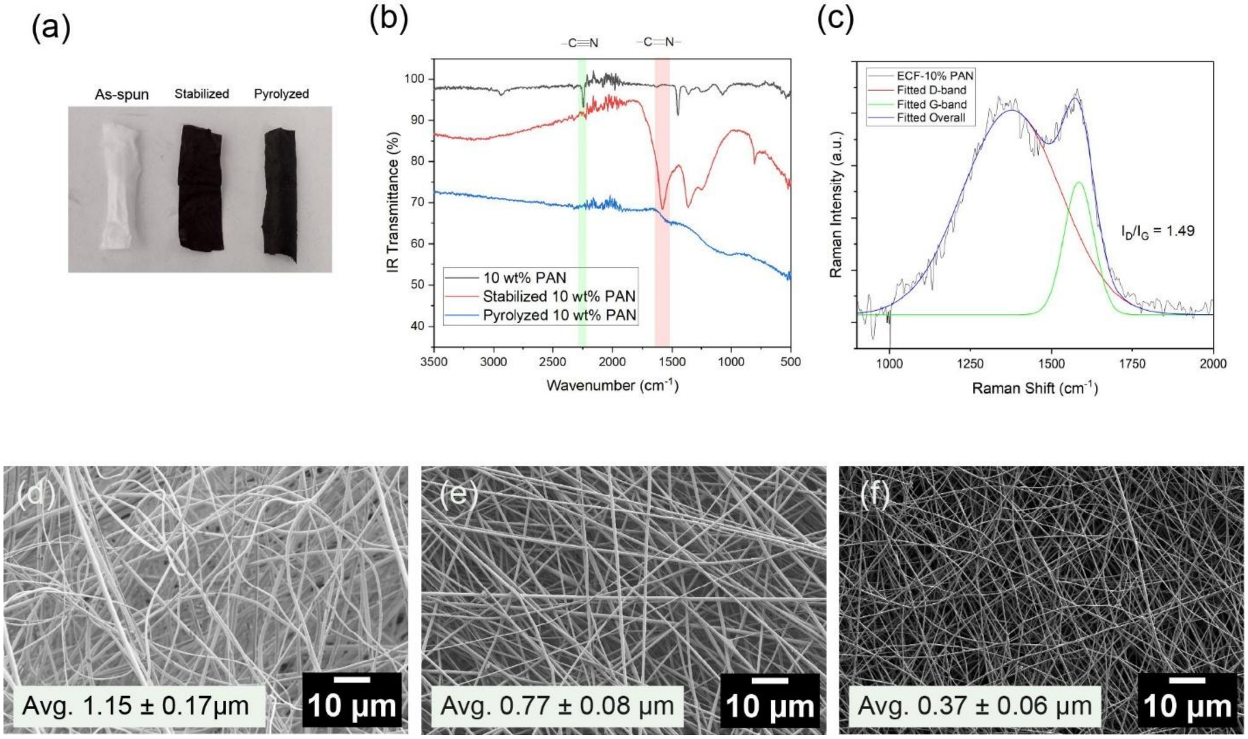

Figure S1.(a) Visual representation of as-spun, stabilized, and pyrolyzed 10 wt% PAN fibers. (b) FTIR spectra of electrospun, stabilized, and pyrolyzed 10 wt% PAN fibers.(c) Raman spectra of pyrolyzed 10 wt% PAN fibers. (d-f) SEM images of as-spun (d), stabilized (e), and pyrolyzed 10 wt% PAN fibers.

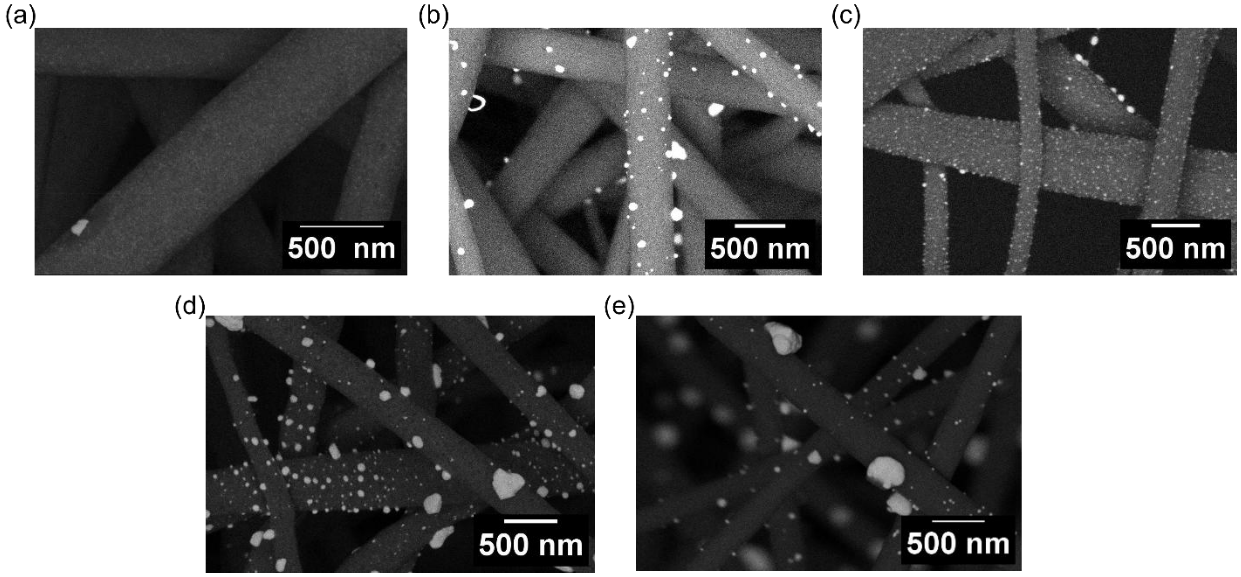

Figure S2. Backscattered SEM images of AgNPs@ECF prepared using $AgNO_3$ concentrations of 1, 3, 5, 7, and 10 mM. Panels (a–e) correspond to 1, 3, 5, 7, and 10 mM, respectively. Images were acquired in backscattered electron (BSE) mode to enhance Z-contrast between Ag nanoparticles and the carbon fiber substrate. Scale bar: 500 nm.

Table S 1. Statistical particle size distribution parameters of AgNPs@ECF prepared using $AgNO_3$ concentrations from 1 to 10 mM, obtained from SEM image analysis.

$AgNO_3$ concentration (mM)	Mean (nm)	Standard deviation	Geometric mean (nm)	GSD	d10 (nm)	d50 (nm)	d90 (nm)	Span
1	15.6	9.56	12.6	1.98	4.07	14.7	27.6	1.60
3	44.2	31.1	33.5	2.28	10.8	41.1	83.5	1.77
5	26.7	19.7	21.9	2.05	9.96	21.9	47.3	1.70
7	36.6	35.4	26.1	2.45	8.09	25.2	75.3	2.66
10	24.5	26.1	16.9	2.33	4.07	17.5	43.6	2.26

d10, d50, and d90 represent particle diameters at 10%, 50%, and 90% cumulative volume, respectively.
Span = (d90 − d10) / d50.

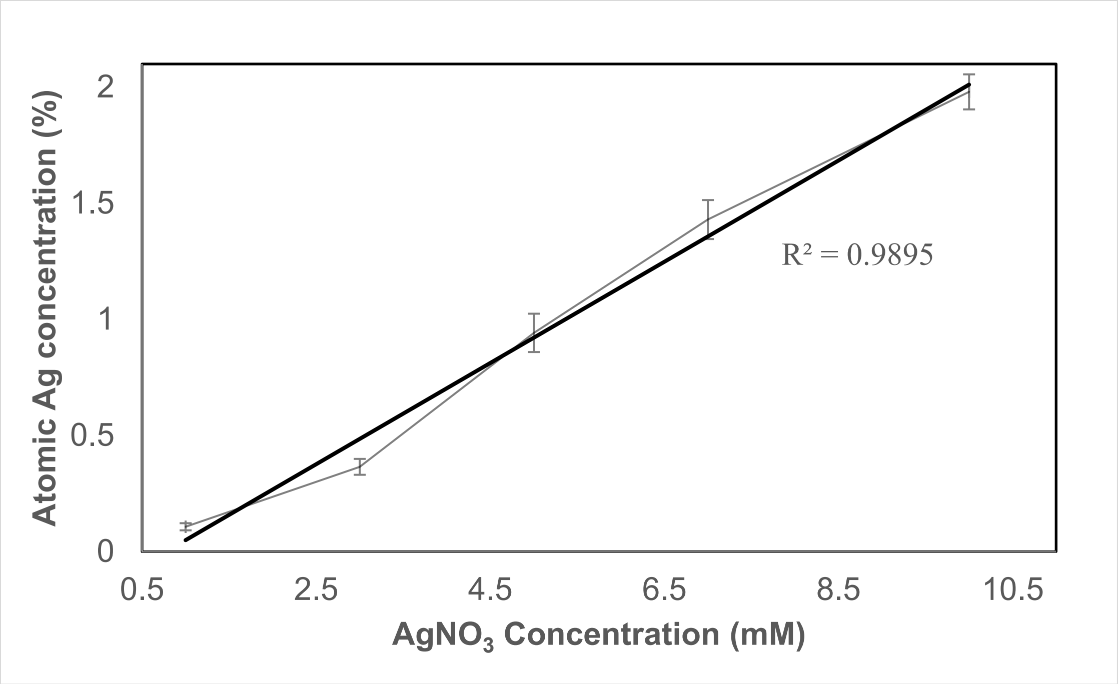

Figure S3. Atomic Ag concentration as a function of $AgNO_3$ precursor concentration. Data points represent mean values with error bars, and the solid line corresponds to a linear fit ($R^2 = 0.9895$).

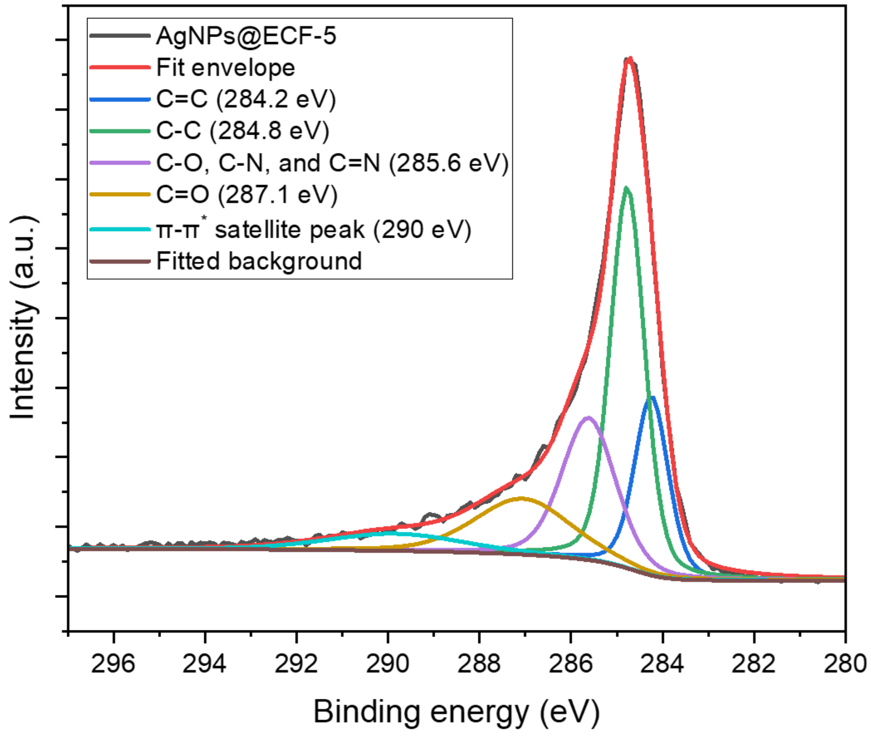

Figure S4. High-resolution C 1s XPS spectrum and peak deconvolution of AgNPs@ECF-5.

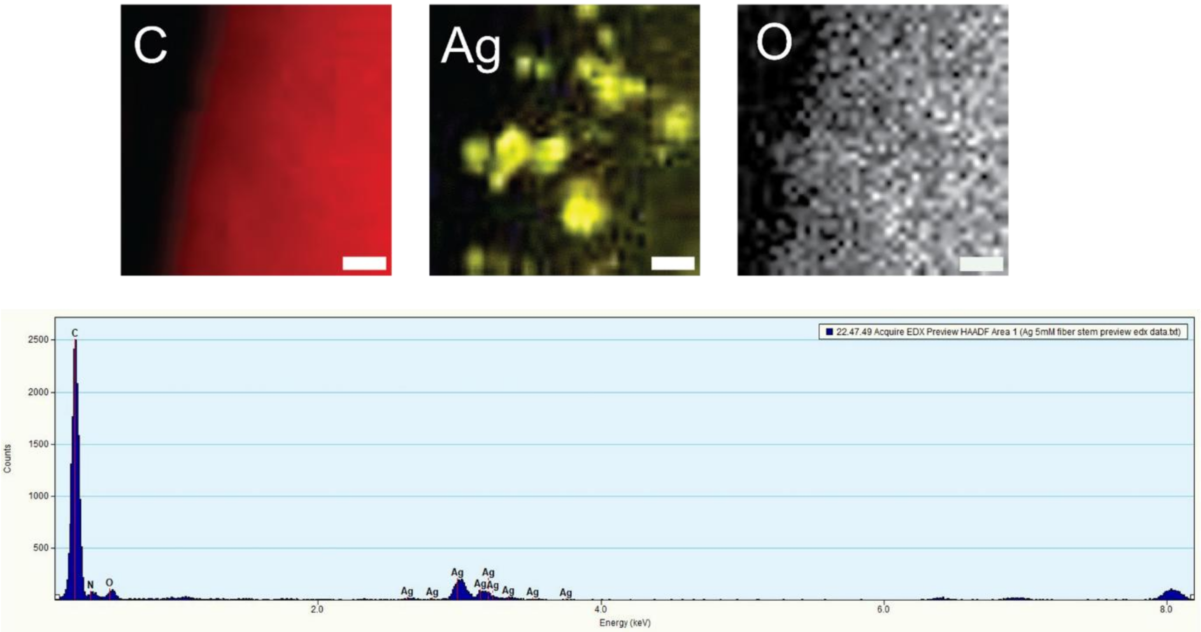

Figure S5. EDS elemental maps and corresponding spectrum of AgNPs@ECF-5, showing uniform carbon distribution and localized Ag nanoparticle domains.

SERS Performance of AgNPs@ECF-5 with 4-MBA molecular probe

SERS enhancement factor (EF) was calculated with the equation:

$$EF = \frac{I_{SERS}}{I_{non-SERS}} x \frac{C_{non-SERS}}{C_{SERS}}$$

where $I_{\mathrm{SERS}}$and $I_{\mathrm{non\text{-}SERS}}$are the Raman intensities obtained from the SERS substrate and the non-enhancing reference substrate, respectively, and $C_{\mathrm{SERS}}$and $C_{\mathrm{non\text{-}SERS}}$are the corresponding analyte concentrations.

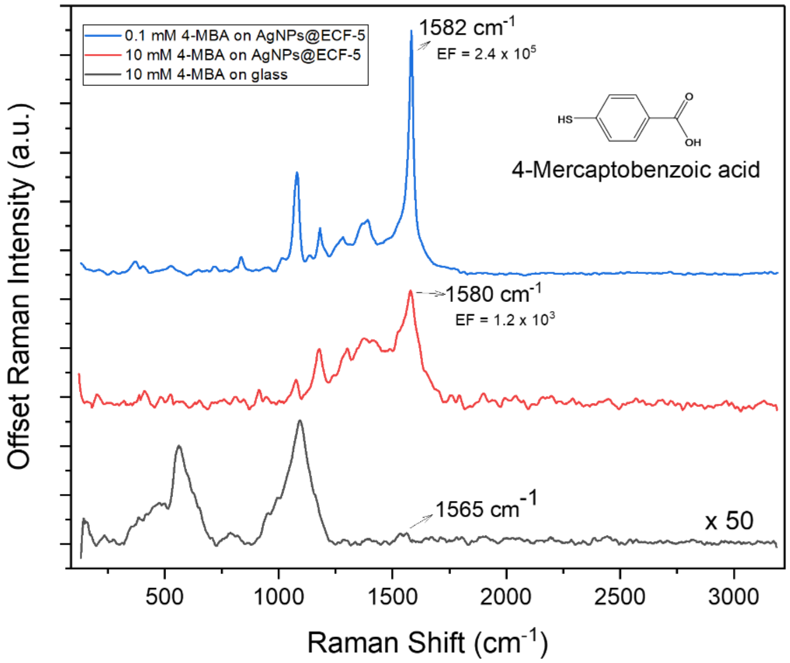

Figure S6. SERS spectra of 4-MBA on AgNPs@ECF-5 at different concentrations, compared with a non-SERS reference on glass. Enhancement factors were calculated using the ~1580 cm $^{-1}$ band.

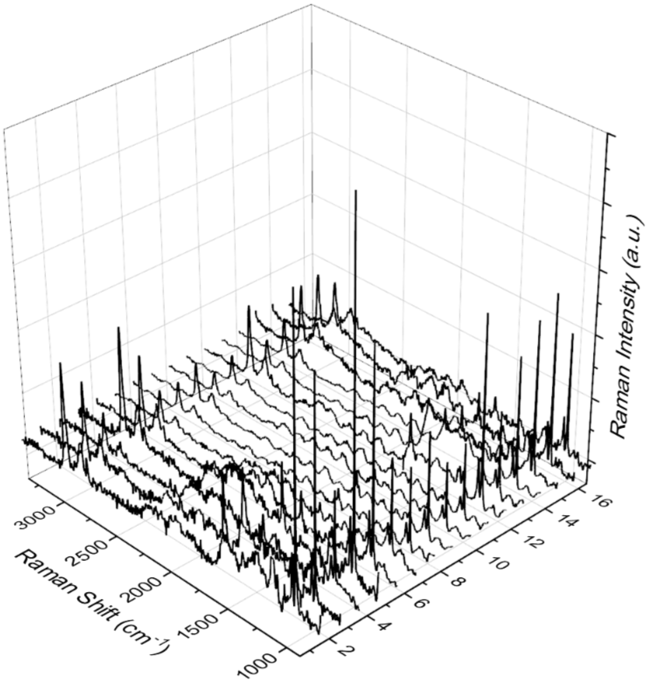

Figure S7. Three-dimensional waterfall plot of SERS spectra collected from multiple locations on AgNPs@ECF-5, demonstrating signal uniformity and reproducibility.

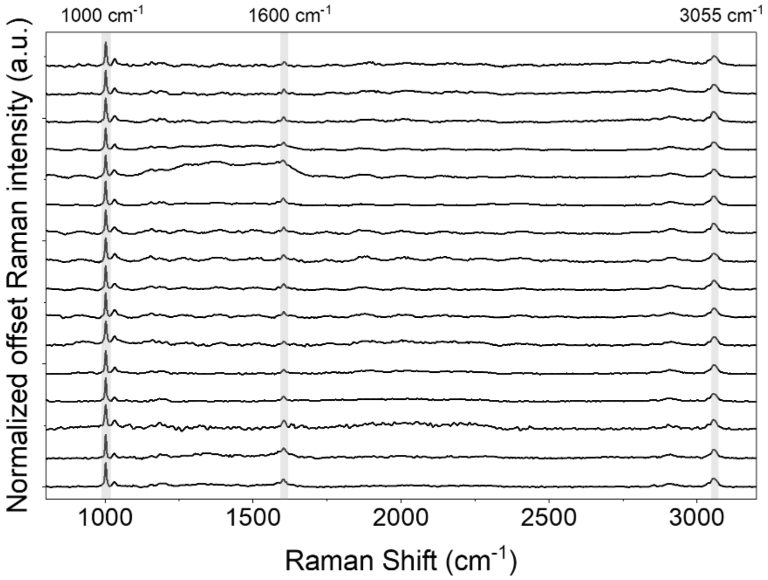

Figure S8. Normalized SERS spectra acquired at different locations on AgNPs@ECF-5.

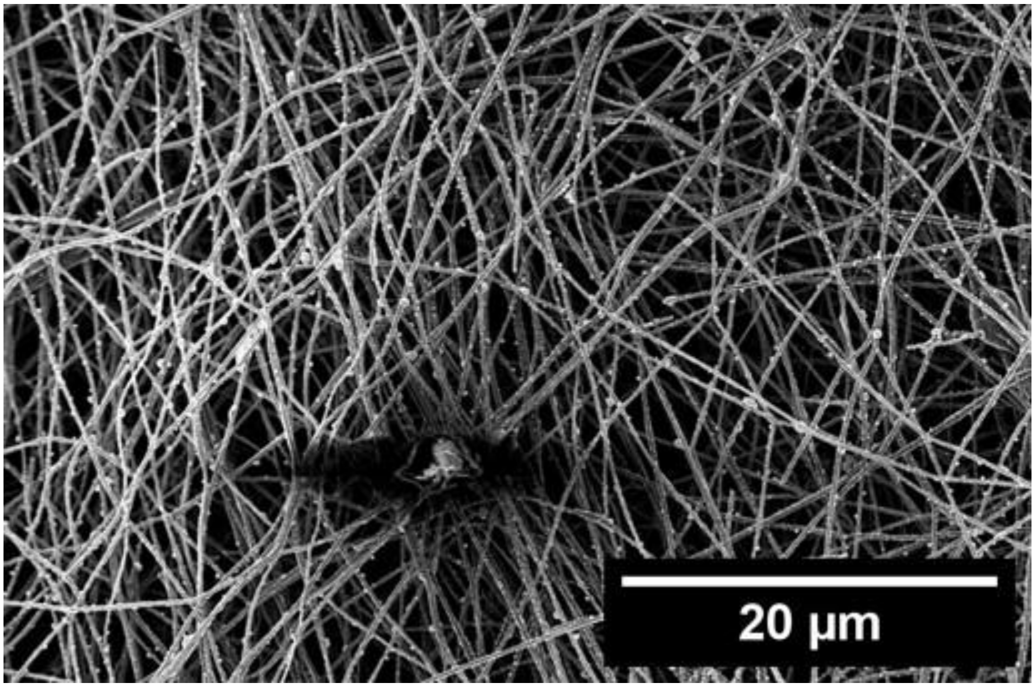

Figure S10. Secondary electron SEM image of AgNPs@ECF-5 in the presence of microplastic particles.

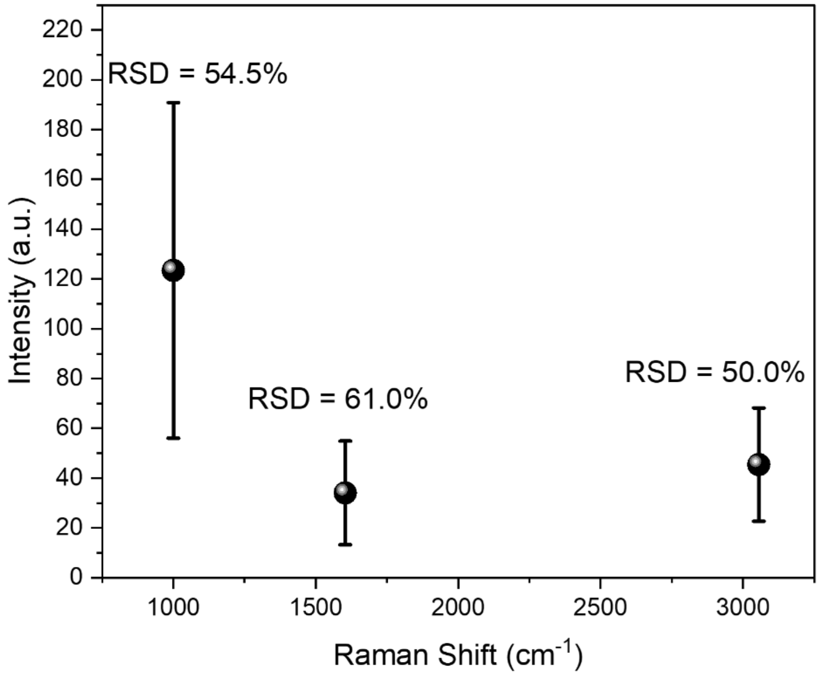

Figure S9. Relative standard deviation (RSD) of SERS intensities at characteristic Raman bands of aged PS, calculated from 16 spectra collected across AgNPs@ECF-5, indicating signal variability across the substrate.

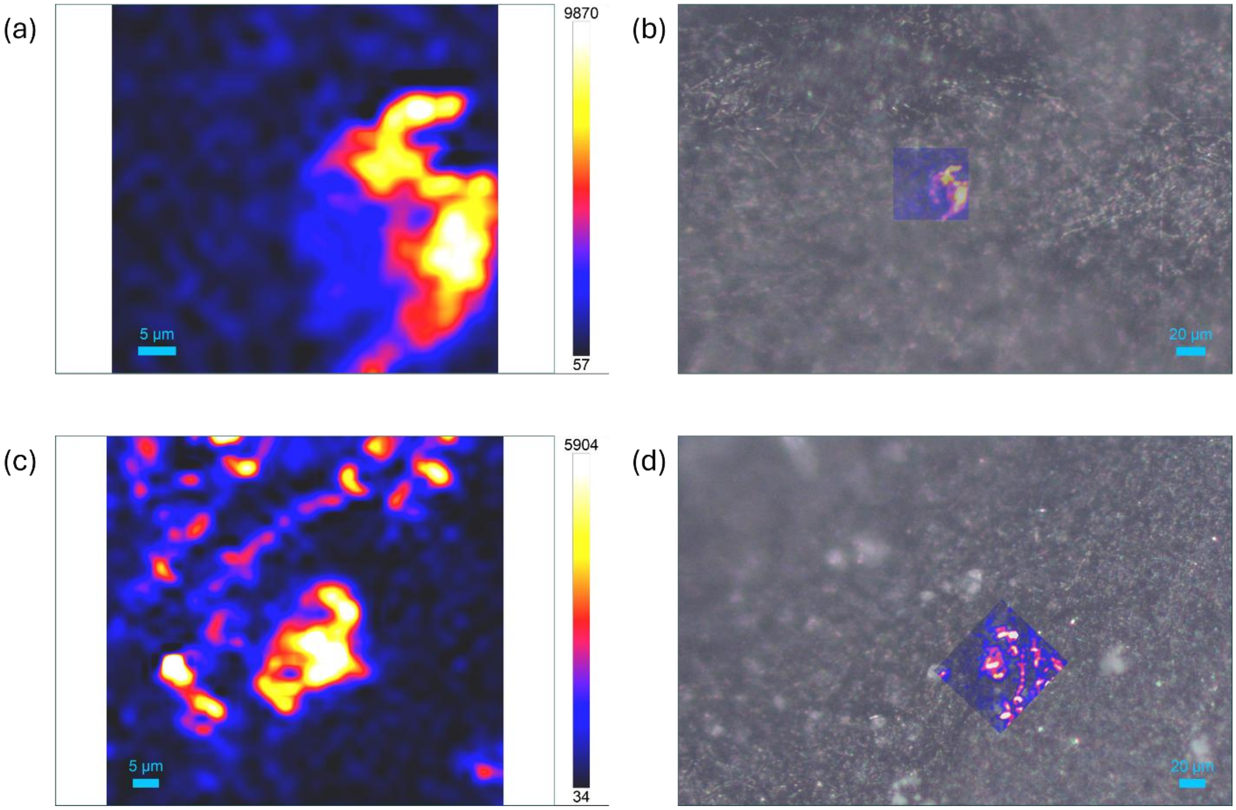

Figure S11. Hyperspectral Raman maps of PET at 1700 cm $^{-1}$ (a) and PS at 1000 cm $^{-1}$ (c), with corresponding optical image overlays (b, d).
